# Accuracy of Flash Glucose Monitoring During Postprandial Rest and Different Walking Conditions in Overweight or Obese Young Adults

**DOI:** 10.3389/fphys.2021.732751

**Published:** 2021-10-15

**Authors:** Xiaoyuan Zhang, Fenghua Sun, Waris Wongpipit, Wendy Y. J. Huang, Stephen H. S. Wong

**Affiliations:** ^1^Department of Sports Science and Physical Education, Faculty of Education, The Chinese University of Hong Kong, Shatin, Hong Kong, SAR China; ^2^School of Sports Medicine and Rehabilitation, Beijing Sport University, Beijing, China; ^3^Department of Health and Physical Education, The Education University of Hong Kong, Tai Po, Hong Kong, SAR China; ^4^Division of Health and Physical Education, Faculty of Education, Chulalongkorn University, Bangkok, Thailand; ^5^Department of Sport, Physical Education, and Health, Hong Kong Baptist University, Kowloon, Hong Kong, SAR China

**Keywords:** obesity, sensor accuracy, exercise, postprandial glycemia, continuous glucose monitoring

## Abstract

**Aims:** To investigate the accuracy of FreeStyle Libre^TM^ flash glucose monitoring (FGM) relevant to plasma glucose (PG) measurements during postprandial rest and different walking conditions in overweight/obese young adults.

**Methods:** Data of 40 overweight/obese participants from two randomized crossover studies were pooled into four trials: (1) sitting (SIT, *n* = 40); (2) walking continuously for 30 min initiated 20 min before individual postprandial glucose peak (PPGP) (20iP + CONT, *n* = 40); (3) walking continuously for 30 min initiated at PPGP (iP + CONT, *n* = 20); and (4) accumulated walking for 30 min initiated 20 min before PPGP (20iP + ACCU, *n* = 20). Paired FGM and PG were measured 4 h following breakfast.

**Results:** The overall mean absolute relative difference (MARD) between PG and FGM readings was 16.4 ± 8.6% for SIT, 16.2 ± 4.7% for 20iP + CONT, 16.7 ± 12.2% for iP + CONT, and 19.1 ± 6.8% for 20iP + ACCU. The Bland–Altman analysis showed a bias of −1.03 mmol⋅L^–1^ in SIT, −0.89 mmol⋅L^–1^ in 20iP + CONT, −0.82 mmol⋅L^–1^ in iP + CONT, and −1.23 mmol⋅L^–1^ in 20iP + ACCU. The Clarke error grid analysis showed that 99.6–100% of the values in all trials fell within zones A and B.

**Conclusion:** Although FGM readings underestimated PG, the FGM accuracy was overall clinically acceptable during postprandial rest and walking in overweight/obese young adults.

## Introduction

Continuous glucose monitoring (CGM), a minimally invasive method of continuously measuring interstitial glucose levels to gain insight into their patterns and trends, has been increasingly adopted as a novel and feasible tool to improve glycemic control in individuals with diabetes and those with poor glucose control. Compared with conventional glucose monitoring, CGM provides much greater insight into glucose levels throughout the day (Klonoff, 2005). As it is now widely used in research, CGM allows for opportunities to continuously examine glycemic control and postprandial glucose (PPG), as well as glucose responses to exercise and meals (Macleod et al., 2013). While CGM has been widely evaluated as a reliable tool for use during resting conditions among individuals with diabetes, the CGM accuracy while exerting effort during common activities such as walking remains controversial (Baek et al., 2010; Biagi et al., 2018; Heden et al., 2018; Moser et al., 2019). Previous studies (Bally et al., 2016; Guillot et al., 2020) and a recent review (Houlder and Yardley, 2018) revealed good and comparable accuracy of the CGM system during different types of exercise, suggesting that current CGM sensors perform at an adequate level to reflect changes in plasma glucose (PG) during exercise in patients with type 1 diabetes mellitus (T1DM); however, PG is still underestimated in CGM readings. A more recent meta-analysis (Fabra et al., 2021) compared the mean absolute relative difference (MARD) during exercise and rest, showing that exercise negatively affects CGM accuracy. Some studies have reported poor performance during rapid glucose changes after a meal (Yan et al., 2020) or during exercise (Biagi et al., 2018; Heden et al., 2018; Moser et al., 2019). One possible reason for this underestimation is the time delay between blood and interstitial fluid compartments. Exercise significantly increases this time delay (Castle and Rodbard, 2019), likely, in part, by stimulating endogenous glucose production (Basu et al., 2013, 2014). Moreover, exercise impacts volume and fluid distribution within the interstitial compartment, contributing to the creation of gradients between the interstitial and blood glucose levels (Moser et al., 2018). However, as few studies have simultaneously reported on CGM accuracy for both exercise and rest periods, it was difficult to examine the influence of exercise on CGM accuracy (Fabra et al., 2021). Therefore, additional studies are warranted to characterize how well current CGM systems perform during exercise (Castle and Rodbard, 2019) and to provide further insight into their accuracy during exercise periods. Notably, glycemic responses to exercise vary according to multiple factors, including exercise timing, intensity, and duration (Riddell et al., 2017). Therefore, additional studies are needed to better explore the performance of CGM systems under different exercise conditions (Forlenza et al., 2019). A better understanding of CGM accuracy under exercise conditions may provide evidence to develop CGM algorithms that may be able to compensate for mitigating its impact (Laguna Sanz et al., 2019).

Continuous glucose monitoring has been widely used in many studies on individuals with diabetes; recently, it has also been increasingly used in studies on healthy volunteers (Basu et al., 2013; DiPietro et al., 2013; Wijsman et al., 2013; Parker et al., 2017; Shah et al., 2019; DuBose et al., 2021). For research purposes, CGM is portable, easy to use, and pain-free, allowing researchers to continuously monitor interstitial glucose levels in daily life. Evaluating CGM performance in healthy populations without diabetes is essential for the interpretation of CGM metrics, as well as for its wider use in research with this population (DuBose et al., 2021). However, limited information exists on CGM-measured glucose concentrations in individuals without diabetes (DuBose et al., 2021), and the validity of CGM-estimated glycemia has not been well-established in this population (Akintola et al., 2015). Although a recent multicenter study (Shah et al., 2019) reported on CGM profiles of healthy participants, a comparison between CGM and blood glucose readings is lacking. Additionally, overweight/obesity is associated with impaired glucose metabolism (Power and Thomas, 2011). A recent study reported that body mass index (BMI) may be associated with CGM accuracy, with CGM underestimating PG in overweight/obese youth without diabetes and overestimating it in those with a lower BMI (Ghane et al., 2019). However, little is known regarding the CGM accuracy during postprandial rest and under different exercise conditions in adults without diabetes who are overweight or obese.

Although a previous study evaluated the accuracy of CGM in healthy populations without diabetes, it did not explore CGM performance following a meal or under different exercise conditions (Akintola et al., 2015). The FreeStyle Libre^TM^ 14-day Flash Glucose Monitoring (FGM) system (FGM, Abbott, Chicago, IL, United States) is a variety of CGM in which the data are available on demand at any time for 14 days; it requires no calibration during its use. The purpose of this study was to evaluate the accuracy of FGM relevant to PG measurements during postmeal rest and different walking conditions in overweight or obese young adults. We hypothesized that FGM accuracy would be clinically acceptable relevant to PG measurements during postmeal rest and walking conditions in this population.

## Materials and Methods

This work is a secondary analysis of two completed studies, both of which are registered at the Chinese Clinical Trial Registry [ChiCTR1900023175 (Zhang et al., 2021); ChiCTR2000035064] and approved by the Joint Chinese University of Hong Kong New Territories East Cluster Clinical Research Ethics Committee (CREC Ref. No.: 2019.285; 2020.434). Written informed consent was obtained from all participants prior to their participation in the study.

### Participants

Individuals who were overweight or obese (BMI ≥ 23.0 kg⋅m^–2^) (WHO Expert Consultation, 2004), aged 18–35 years, had a self-reported daily sedentary time ≥8 h, and insufficient physical activity (PA) (engaging in < 150 min of moderate-intensity PA per week or < 75 min of vigorous-intensity PA per week over the past 3 months, as measured using the International Physical Activity Questionnaire Short Form) were included in this study. Exclusion criteria included hyperglycemia (fasting blood glucose higher than 7.1 mmol⋅L^–1^), a chronic disease diagnosis, blood pressure >130/80 mmHg, using any medication known to affect lipid or glucose metabolism, alcohol/drug abuse, smoking, or having a musculoskeletal injury impairing their ability to walk or run on a treadmill. A total of 40 participants completed the 2 studies.

### Study Design and Protocols

Data were pooled from two randomized crossover studies. Participants remained seated or walked for 30 min at 50% V̇O_2m__ax_ on a treadmill following a meal; differently timed walking or patterns were implemented in each trial. Specifically, 20 participants underwent three randomly ordered trials in study 1 (Zhang et al., 2021): (1) SIT: sitting for 240 min; (2) 20iP + CONT: postmeal walking continuously for 30 min initiated 20 min before individualized postprandial glucose peak time (PPGP); and (3) iP + CONT: postmeal walking continuously for 30 min initiated at individualized PPGP; in study 2, the remaining 20 participants underwent another three randomly ordered trials: (1) SIT: sitting for 240 min; (2) 20iP + CONT: postmeal walking continuously for 30 min initiated 20 min before individualized PPGP; and (3) 20iP + ACCU: accumulated postmeal walking for 30 min initiated 20 min before individualized PPGP (three bouts of 10 min walking separated by 20 min of rest). A 6–14-day washout period was allowed between trials. Since SIT and 20iP + CONT were the same trials in both studies, we combined these data for further analyses.

#### Screening Visit

During the screening visit, height, body mass, and body fat percentage were measured. BMI was calculated as body mass (kg) divided by height squared (m^2^). Capillary blood samples were collected to assess fasting glucose. Each participant’s V̇O_2m__ax_ and the speed equivalent to 50% V̇O_2m__ax_ were tested as reported previously (Zhang et al., 2021).

#### Postprandial Glucose Peak Determination

Participants wore a factory calibrated FGM (FreeStyle Libre, Abbott) sensor to monitor interstitial glucose. The FGM sensor was inserted under the skin on the back of the upper arm at least 24 h before testing. Participants were instructed to monitor their 2-h postprandial interstitial glucose every 5 min for three consecutive days when sitting in the laboratory to determine their PPGP in response to the same breakfast as in the main trial (Zhang et al., 2021). Individualized PPGP was determined as the average over 3 days. During free-living conditions, participants were asked to scan the sensor using a reader at least every 8 h to store their glucose readings.

#### Main Trials

On the main trial day, participants reported to the laboratory after fasting overnight for 12 h. A cannula was inserted into any accessible forearm vein for repeated venous blood sample collection. After 1 h of resting, 20 participants consumed white bread, containing 1 g of carbohydrates per kg of body mass (73% carbohydrates, 12% protein, and 15% fat) in study 1 (Zhang et al., 2021); in study 2, 20 participants consumed a standardized mixed meal, providing 33% of the individual daily energy requirements estimated using the Mifflin-St Jeor equation (Mifflin et al., 1990), with a PA factor of 1.4 (65% carbohydrates, 10% protein, and 25% fat). A 280-mL bottle of water was provided during breakfast. Participants were instructed to consume the meal within 10 min. Although the SIT and 20iP + CONT groups, whose data were pooled for this secondary analysis, consumed different meals in the two studies, this did not have a distinct impact on FGM sensor performance (similar MARD results, details not shown).

Following the commencement of eating (set as 0 min), participants either remained seated for 240 min (SIT), walked on a treadmill for 30 min, beginning 20 min before their individualized PPGP (20iP + CONT), walked for 30 min, beginning at PPGP (iP + CONT), or walked for three bouts of 10 min, separated by 20 min of resting, beginning 20 min prior to PPGP (20iP + ACCU). All walking was performed at 50% V̇O_2m__ax_. After walking, participants sat (reading, playing games, watching TV, computer working, etc.) for 240 min. During walking, ratings of perceived exertion (Borg, 1982) were recorded and the heart rate was measured every 5 min using a heart rate monitor.

#### Measurement

Throughout each trial, a 3-mL blood sample was collected into a K_2_EDTA tube every 15 and 30 min for 0–120 and 120–240 min, respectively. PG concentrations were measured using an EKF Biosen C-Line glucose analyzer (EKF Diagnostic, Barleben, Germany) *via* the glucose oxidase method (Nowotny et al., 2012). The intra-assay coefficient of variation for PG was 0.2%. FGM data were monitored within 1 min of blood collection.

Participants were instructed to avoid any planned exercise for 72 h and to refrain from alcohol and caffeine consumption for 48 h before the main trials; this was confirmed verbally upon arrival. For screening, participants were asked to wear the activPAL3 micro accelerometer (PAL Technologies, Glasgow, United Kingdom) for 7 days, as well as for 48 h before each main trial; PA levels were assessed at these points to ensure similar PA between trials. Participants recorded all food and liquid intake in a food diary for 48 h prior to the first experimental condition, with diets then replicated prior to each subsequent main trial. For all trials, participants came to the laboratory using the same public transportation.

### Statistical Analysis

Data are reported as mean ± standard deviation (SD) and analyzed using SPSS (Version 23.0, IBM Corp., Armonk, NY, United States). FGM sensor performance was analyzed using the MARD, Pearson’s correlations, and the Bland–Altman method [bias and 95% limits of agreement (LoA)]. The MARD was calculated using the formula | FGM glucose—PG| /PG, to assess FGM accuracy (Akintola et al., 2015). A comparison of FGM and PG readings was conducted using paired *t*-tests. One-way repeated-measures analysis of variance (ANOVA) was used to compare the overall MARD between trials (factor: intervention), as well as to compare the MARD between time points in each trial (factor: time). Accuracy metrics included the proportion of FGM system values that were within ±20% of the paired PG values >100 mg⋅dL^–1^ or ±20 mg⋅dL^–1^ of the PG values ≤100 mg⋅dL^–1^ (hereafter referred to as %20/20), as well as the analogous %15/15 and %30/30. Bland–Altman plots (Bland and Altman, 1986) were used to depict data distribution and bias between the FGM and PG values by plotting the differences between values from each measurement method at each time point against the average of the two methods. Intraclass correlation coefficient (ICC) estimates and their 95% confident intervals (CI) were calculated based on single measure, absolute-agreement, two-way mixed-effects model. Clarke error grid analysis (Clarke et al., 1987) and Parkes error grid analysis (Parkes et al., 2000) were performed to quantify the clinical accuracy of FGM using MATLAB R2019b (The MathWorks, Inc., Natick, MA, United States). In these analyses, five zones characterize errors of varied clinical significance; values in zones A and B are defined as “clinically acceptable,” while those in zones C, D, or E are considered potentially unsafe, likely leading to clinically significant errors. The percentage of data points in each zone was calculated (Wentholt et al., 2008). A *P*-value < 0.05 was accepted as statistically significant.

## Results

### Participant Characteristics

Forty participants (31 males) aged 22.8 ± 4.2 years, with a BMI of 28.5 ± 3.3 kg⋅m^–2^ and a V̇O_2m__ax_ of 29.6 ± 7.4 mL⋅min^–1^⋅kg^–1^, were involved in this study. All participants were within the normal range for fasting glucose (5.0 ± 0.4 mmol⋅L^–1^) and glycated hemoglobin (5.3 ± 0.4%). The determined PPGP ranged from 36 to 67 min (49.7 ± 8.4 min). Participants started walking at an average of 30 min (16–47 min) in 20iP + CONT and 20iP + ACCU and an average of 50 min (36–67 min) in iP + CONT.

### Accuracy of Flash Glucose Monitoring

**FIGURE 1:**
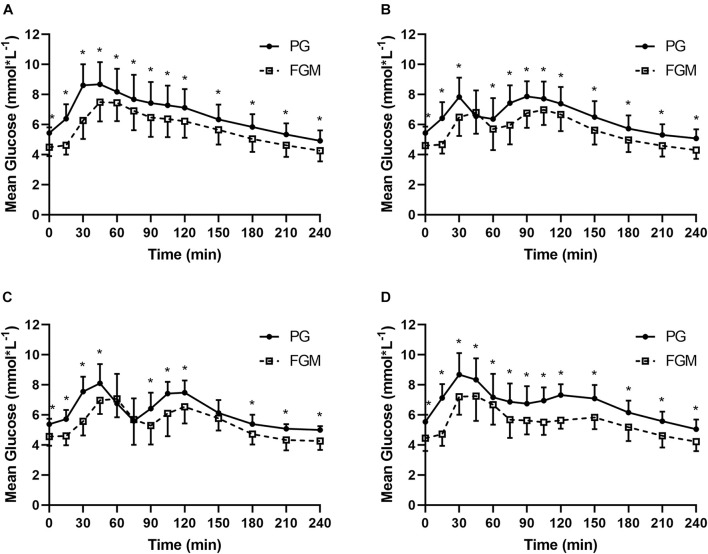
Glucose values from PG (full line) and FGM (dotted line) during SIT **(A)**, 20iP + CONT **(B)**, iP + CONT **(C)**, and 20iP + ACCU **(D)**. PG, plasma glucose; FGM, interstitial glucose monitored by FGM; SIT, sitting. 20iP + CONT: walking continuously for 30 min initiated 20 min before each participant’s postprandial glucose peak (PPGP); iP + CONT: walking continuously for 30 min initiated at PPGP; and 20iP + ACCU: walking accumulated for 30 min initiated 20 min before PPGP (three bouts of 10 min walking separated by 20 min of rest). Data represent mean ± standard deviation (SD). Paired *t*-test was used for comparison between FGM and PG. *P < 0.05, PG vs. FGM.

There were 520 pairs of FGM and PG values in SIT (*n* = 40), 519 pairs in 20iP + CONT (*n* = 40), 258 pairs in iP + CONT (*n* = 20), and 259 pairs in 20iP + ACCU (*n* = 20). Except for 45 min in 20iP + CONT, and 60, 75, and 150 min in iP + CONT, the FGM readings were lower than the measured PG at all time points (all *P* < 0.05). No differences in the overall MARD were found between trials (*P* = 0.465). Readings and the MARD of PG and FGM during SIT and 20iP + CONT are illustrated in Supplementary Table 1 and Figure 1. The overall %20/20, %15/15, and %30/30 accuracies are shown in Supplementary Table 1.

**FIGURE 2:**
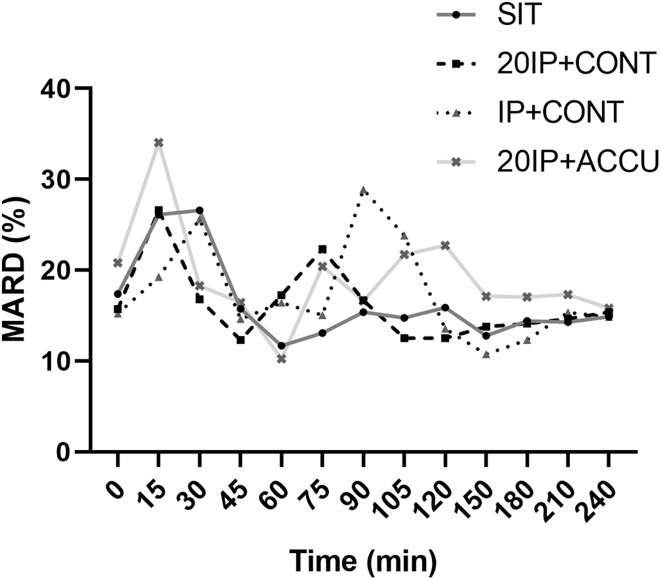
MARD under postprandial resting and different walking conditions. MARD, mean absolute relative difference; SIT, sitting. 20iP + CONT: walking continuously for 30 min initiated 20 min before each participant’s postprandial glucose peak (PPGP); iP + CONT: walking continuously for 30 min initiated at PPGP; and 20iP + ACCU: walking accumulated for 30 min initiated 20 min before PPGP (three bouts of 10 min walking separated by 20 min of rest). One-way repeated-measures ANOVA (factor: time) was used for the comparison of MARD between time points in each trial.

In SIT, the MARD was higher at 15 and 30 min than at any other time point (*P* < 0.05), while in 20iP + CONT, it was higher at 15 min than at any other time point (*P* < 0.05); The MARD was also higher at 75 min than at 105–240 min. In iP + CONT, the MARD was higher at 30 min than at 45 and 120–240 min (*P* < 0.05). In 20iP + ACCU, the MARD was higher at 15 min than at any other time point and was lower at 60 min than at 0, 105, 120, and 240 min (*P* < 0.05) (Figure 2).

### Pearson Correlation

A strong positive correlation was found between FGM and PG in all four trials (*r* = 0.775, *P* < 0.001 in SIT; *r* = 0.778, *P* < 0.001 in 20iP + CONT; *r* = 0.673, *P* < 0.001 in iP + CONT; and *r* = 0.765, *P* < 0.001 in 20iP + ACCU).

### Bland–Altman Method and Intraclass Correlation Coefficient

**FIGURE 3:**
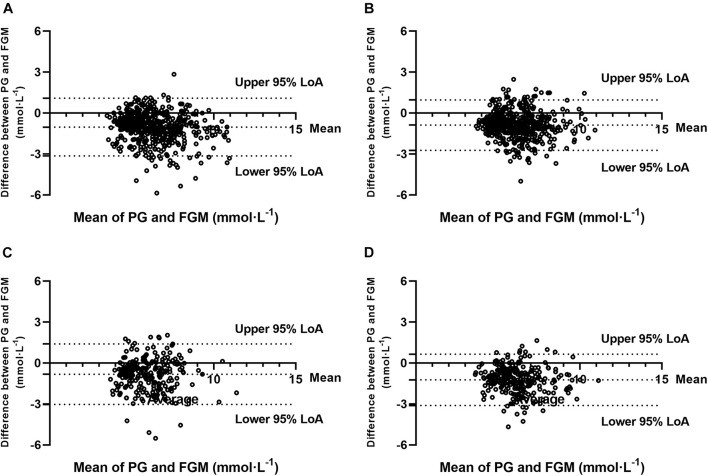
Bland-Altman plots for the comparison of PG and FGM values during **(A)** SIT, **(B)** 20iP + CONT, **(C)** iP + CONT, and **(D)** 20iP + ACCU. 95% LoA, 95% Limit of Agreement; PG, plasma glucose; FGM, interstitial glucose monitored by FGM; SIT, sitting. 20iP + CONT: walking continuously for 30 min initiated 20 min before each participant’s postprandial glucose peak (PPGP); iP + CONT: walking continuously for 30 min initiated at PPGP; and 20iP + ACCU: walking accumulated for 30 min initiated 20 min before PPGP (three bouts of 10 min walking separated by 20 min of rest).

The Bland–Altman method-derived bias and LoAs for absolute values of glucose (FGM to PG) were found at −1.03 (−3.15, 1.09) mmol⋅L^–1^ in SIT, −0.89 (−2.74, 0.96) mmol⋅L^–1^ in 20iP + CONT, −0.82 (−3.04, 1.39) mmol⋅L^–1^ in iP + CONT, and −1.23 (−3.12, 0.65) mmol⋅L^–1^ in 20iP + ACCU. Furthermore, 94.4% (491/520), 92.5% (480/519), 95.7% (247/258), and 93.4% (242/259) pairs of values were within the 95% LoA in SIT, 20iP + CONT, iP + CONT, and 20iP + ACCU, respectively (Figure 3). The obtained ICC indicated a moderate reliability between PG and FGM measurements (ICC = 0.637, 95% CI [0.120, 0.825], *P* < 0.001 in SIT; ICC = 0.648, 95% CI [0.137, 0.831], *P* < 0.001 in 20IP + CONT; ICC = 0.574, 95% CI [0.229, 0.748], *P* < 0.001 in IP + CONT; and ICC = 0.550, 95% CI [−0.062, 0.802], *P* < 0.001 in 20IP + ACCU).

### Clarke Error Grid Analysis

**FIGURE 4:**
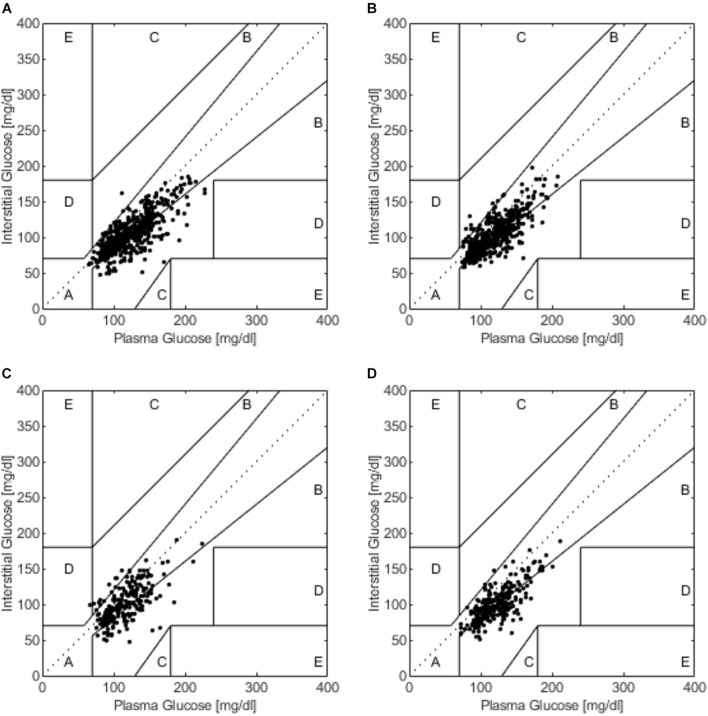
The Clarke error grid analysis during **(A)** SIT, **(B)** 20iP + CONT, **(C)** iP + CONT, and **(D)** 20iP + ACCU. SIT, sitting. 20iP + CONT: walking continuously for 30 min initiated 20 min before each participant’s postprandial glucose peak (PPGP); iP + CONT: walking continuously for 30 min initiated at PPGP; and 20iP + ACCU: walking accumulated for 30 min initiated 20 min before PPGP (three bouts of 10 min walking separated by 20 min of rest).

Clarke error grid analysis showed that 100% of values were within zones A and B in SIT, with 70.8% in zone A and 29.2% in zone B. During 20iP + CONT, 100% of values were in zones A and B, with 69.4% in zone A and 30.6% in zone B. During iP + CONT, 99.6% of values were in zones A and B, with 67.1% in zone A, 32.6% in zone B, and 0.4% in zone D. During 20iP + ACCU, 100% of values were in zones A and B, with 54.8% in zone A and 45.2% in zone B (Figure 4). In addition, the results of Parkes error grid analysis (Parkes et al., 2000; Supplementary Figure 1), which advantageously shows more continuous transitions between adjacent zones, revealed similar results to those in the Clarke error grid analysis.

### Regression Analysis

The regression analyses were performed on several key time points (at fasting state, immediately after a meal, at PPGP, and at 60 min after a meal) to serve a greater purpose for FGM users, especially researchers, by generating a regression-generated equation to correct FGM values. The equations were provided in Supplementary Figure 2 and Supplementary Table 2.

## Discussion

This work was the first comparison of techniques for FGM vs. PG monitoring under postprandial rest and different walking conditions among overweight or obese adults. Although FGM readings were significantly lower than those of PG during both postmeal sitting and walking in overweight or obese adults, a strong correlation was observed between FGM and PG, indicating that FGM could depict patterns of PG change. Furthermore, the MARD was higher when glucose rapidly increased after a meal or walking The FGM sensor performed well, showing stability when the glucose level was stable; timing and patterns of walking did not affect sensor performance. Moreover, the overall FGM performance was clinically acceptable, as shown through Clarke error grid analysis and the Bland–Altman method.

Continuous glucose monitoring readings have been reported to be lower than PG (Yardley et al., 2013; Yan et al., 2020). Consistently, FGM readings were 0.8–1.2 mmol⋅L^–1^ lower than PG during postmeal sitting and walking conditions in the present study (Supplementary Table 1). A possible reason for this difference may be blood-to-interstitial glucose kinetics resulting in a time difference between venous blood and interstitial fluid (Lunn et al., 2011; Akintola et al., 2015). In the present study, PPGP showed a 15 min time delay using FGM in relation to using PG. This time lag may be overestimated, as glucose levels were measured at 15 min intervals. Previous studies have reported a 4–13 min time difference using different CGM systems in individuals with T1DM (Boyne et al., 2003; Wilson et al., 2007) and T2DM (Keenan et al., 2012).

However, as the FGM values were lower than the measured PG even when the glucose levels were steady in the fasted state, the underestimation of glucose by FGM could be a systematic sensor characteristic, rather than a physiological time delay between interstitial glucose and PG (Figure 1). Moreover, considerable inter-sensor variability was observed in a previous study using Medtronic/MiniMed CGM (Boyne et al., 2003), with the response characteristics of this glucose sensor system posing another reason for the observed PPGP time differences between FGM and PG. Interestingly, a recent study (Ghane et al., 2019) reported that BMI, total body fat mass, and fat percentage were similarly associated with CGM accuracy, suggesting that differences in subcutaneous fat may account for the changes in CGM accuracy in overweight/obese individuals. In contrast, Massa et al. (2018) and Ghane et al. (2019) reported that CGM overestimates glucose in individuals with a lower BMI. Furthermore, CGM overestimated blood glucose during a 30-min bout of high-intensity cycling performed 2 h postmeal in individuals with T1DM (Radermecker et al., 2013). Therefore, CGM accuracy may partly depend on participant characteristics, especially body composition.

The MARD in the present study was acceptable and comparable to that in previous studies. MARDs of 9.2% (Staal et al., 2018), 10.0% (Ji et al., 2017), and 11.4% (Bailey et al., 2015) during free-living conditions and of 13.2% (Bally et al., 2016) and 22.0% (Moser et al., 2019) during exercise were reported in populations with diabetes. Biagi et al. (2018) reported MARDs of 9.5, 16.5, and 9.3% before, during, and after aerobic exercise, respectively, in individuals with T1DM. Notably, the MARD is inflated in normal populations but not in populations with diabetes as its calculation is dependent on the glucose level, which is lower in populations without diabetes. In the present study, the MARD increased both after a meal and soon after walking (Figure 2), which may have been caused by a time delay. PG rapidly increased 15–30 min after starting a meal and after walking (Figure 1), while increases in interstitial glucose were delayed; interstitial glucose was lower than PG, especially in the fasting state (i.e., hypoglycemia for FGM readings) (Figure 1). However, the MARD decreased during walking in the present study (Figure 2). Similarly, Yardley et al. (2013) reported that CGM underestimated PG to the greatest extent during rest (−1.29–1.39 mmol⋅L^–1^, *P* < 0.001), and least during aerobic walking (−0.11–1.71 mmol⋅L^–1^, *P* = 0.416). The superior accuracy observed with aerobic walking may arise from augmented blood flow better equilibrating plasma and interstitial fluid or from a combination of systematic sensor underestimation and sensor lag time.

Overall, we observed no significant differences in the MARD between the postmeal sitting and walking conditions among overweight or obese young adults. In the present study, the %20/20 accuracy in the postprandial resting and walking conditions appeared lower than those reported by previous studies conducted in daily life among populations with diabetes (Shah et al., 2018; Wadwa et al., 2018; Zhou et al., 2018). Walking timing did not affect FGM accuracy. The MARD and %20/20 accuracy were slightly higher and lower, respectively, during accumulated walking, compared with those in the other three trials, potentially because it extended walking duration. As glucose variation increased, the MARD varied during the three bouts of walking.

In the present study, the Bland–Altman method and the Clarke error grid analysis showed that the FGM values were clinically acceptable (Figures 3, 4). In previous studies, almost 100% of the value pairs fell within zones A and B of the Clarke error grid analysis (Bally et al., 2016; Moser et al., 2016; Biagi et al., 2018; Yan et al., 2020). This excellent, clinically acceptable accuracy proved that the CGM accuracy during postmeal resting and walking was high and was further verified using the Bland–Altman method and Pearson’s correlation analysis.

As these findings confirm the clinical stability of FGM under postmeal sitting and walking conditions in overweight or obese young adults, it can be used as an efficient tool for collecting glucose measurements for clinical investigations. However, absolute values should be used with caution when controlling target blood glucose levels. The results of this study may provide a basis from which to develop models to estimate blood glucose levels using interstitial fluid glucose levels obtained *via* CGM systems (Castle and Rodbard, 2019), also establishing compensation schemes to correct the impact of exercise on CGM accuracy (Laguna Sanz et al., 2019). CGM accuracy should be validated during different exercise conditions in both populations with and without diabetes. As the FreeStyle Libre FGM system is indicated for the management of diabetes in persons aged 18 years and older, its performance requires validation in individuals without diabetes. As hypoglycemia is a common concern for individuals with diabetes during exercise, our findings provide some understanding of postmeal exercise and the combined effects of meal and exercise on CGM accuracy.

This study had several strengths. Both FGM and PG data were collected in two well-controlled randomized crossover studies. Several walking conditions with different timing and patterns were compared. Limited information exists regarding FGM accuracy in healthy individuals during different walking conditions. As the timing of walking initiation was individualized based on individualized PPGP, it allowed us to visualize the FGM accuracy during periods of large glucose level variation. With different postmeal walking timing and patterns, the combined effects of meal and walking on FGM accuracy could be observed. Although many previous studies failed to compare MARD values between exercise and rest periods (Fabra et al., 2021), the present study examined the CGM accuracy in a wide variety of conditions. In addition, the present study was conducted among healthy individuals who were overweight or obese, providing more information for this particular population. Venous blood, from which PG was measured as a reference, was collected; this offers more accurate data than those based on capillary glucose used in previous studies. Arterialized-venous samples were previously used as reference measurements, showing no significant impact on CGM accuracy (Kropff et al., 2017); therefore, this reference seems preferable because of its ease of operation.

A limitation of this study was its relatively small sample size (*n* = 40), but several rest and walking conditions were examined using a randomized crossover design. Furthermore, as we only recruited overweight or obese young adults and used a FreeStyle Libre FGM sensor, the generalizability of our findings may be limited. Further studies are needed to recruit diabetic participants with a large sample size.

## Conclusion

Although absolute glucose readings derived using FGM underestimated PG, the overall accuracy of FGM was clinically acceptable during postprandial sitting and walking conditions in overweight or obese young adults.

## Data Availability Statement

The raw data supporting the conclusions of this article will be made available by the authors, without undue reservation.

## Ethics Statement

The studies involving human participants were reviewed and approved by the Joint Chinese University of Hong Kong-New Territories East Cluster Clinical Research Ethics Committee. The patients/participants provided their written informed consent to participate in this study.

## Author Contributions

XZ, FS, and SW designed the research. XZ and WW conducted the research. XZ analyzed the data and drafted the manuscript. FS, WW, WH, and SW made major contributions in revising the manuscript. SW had the primary responsibility for final content. All authors read and approved the final manuscript.

## Conflict of Interest

The authors declare that the research was conducted in the absence of any commercial or financial relationships that could be construed as a potential conflict of interest.

## Publisher’s Note

All claims expressed in this article are solely those of the authors and do not necessarily represent those of their affiliated organizations, or those of the publisher, the editors and the reviewers. Any product that may be evaluated in this article, or claim that may be made by its manufacturer, is not guaranteed or endorsed by the publisher.
