# Supplementary material for: Accuracy of Flash Glucose Monitoring During Postprandial Rest and Different Walking Conditions in Overweight or Obese Young Adults
Source: Front Physiol. 2021 Oct 15;12:732751. doi: 10.3389/fphys.2021.732751 (PMC8555657; doi:10.3389/fphys.2021.732751)
Supplement: Supplementary file 1 [file Data_Sheet_1.docx]

Supplemental Figure 2. Regression equations in fasting state (A), immediately after a meal (B), at PPGP (C), and at 60 min after a meal (D). N = 120 for each time point.


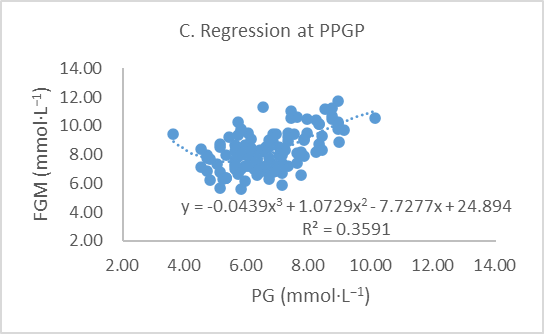

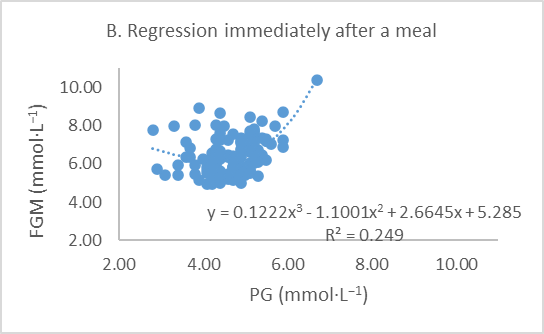

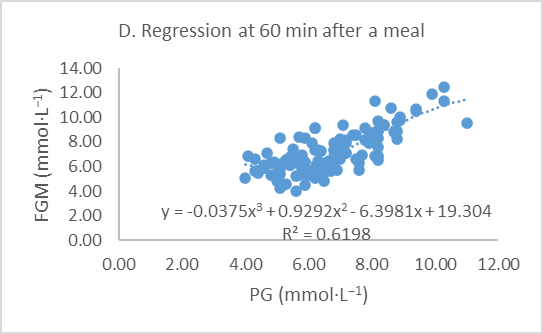

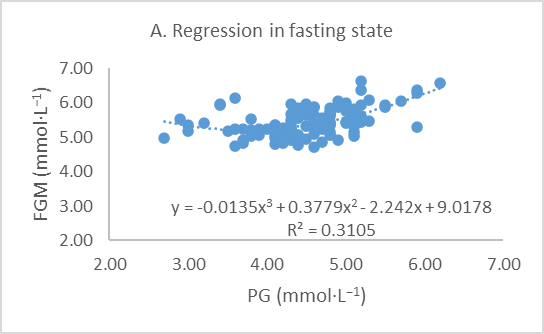


PG, plasma glucose; FGM, interstitial glucose monitored by FGM; PPGP, postprandial glucose peak.

Supplemental Table 2. Regression equations in fasting state, immediately after a meal, at PPGP, and at 60 min after a meal.

| Time point | Fasting state | Immediately after a meal | At postprandial glucose peak | At 60 min after a meal |
| --- | --- | --- | --- | --- |
| Regression equation | y = -0.0135x^3^ + 0.3779x^2^ - 2.242x + 9.0178 | y = 0.1222x^3^ - 1.1001x^2^ + 2.6645x + 5.285 | y = -0.0439x^3^ + 1.0729x^2^ - 7.7277x + 24.894 | y = -0.0375x^3^ + 0.9292x^2^ - 6.3981x + 19.304 |
| Notes | y = interstitial glucose monitored by FGM; x = plasma glucose | | | |
