# Supplementary material for: Accuracy of Flash Glucose Monitoring During Postprandial Rest and Different Walking Conditions in Overweight or Obese Young Adults
Source: Front Physiol. 2021 Oct 15;12:732751. doi: 10.3389/fphys.2021.732751 (PMC8555657; doi:10.3389/fphys.2021.732751)
Supplement: Supplementary file 2 [file Image_1.pdf]

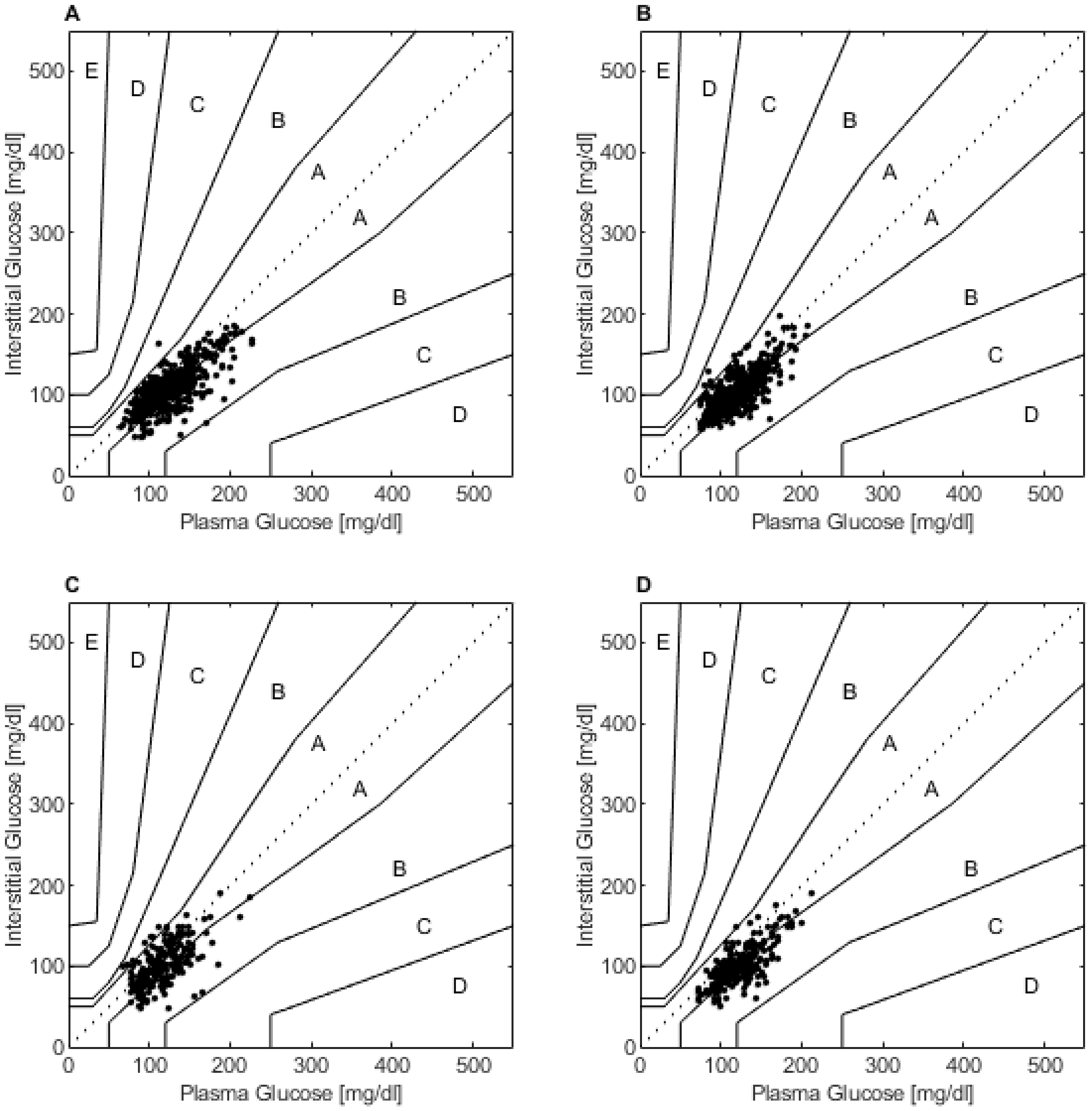


Supplemental Figure 1. Parkes error grid analysis in SIT (A), 20iP+CONT (B), iP+CONT (C), and 20iP+ACCU (D). SIT, sitting; 20iP+CONT: walking continuously for 30 min initiated 20 min before each participant's postprandial glucose peak (PPGP); iP+CONT: walking continuously for 30 min initiated at PPGP; 20iP+ACCU: walking accumulated for 30 min initiated 20 min before PPGP (3 bouts of 10 min walking separated by 20 min of rest).
